# Nguyen Trong Lan, MD, PhD

**DOI:** 10.4269/ajtmh.18-0695

**Published:** 2018-10-22

**Authors:** Nguyen Thanh Hung, Scott B. Halstead

**Affiliations:** 1 Director, Children’s Hospital 1, Ho Chi Minh City, Vietnam;; 2 Department of Preventive Medicine, Uniformed Services University of the Health Sciences, Bethesda, Maryland

Nguyen Trong Lan, MD, PhD, former Head, Dengue Hemorrhagic Fever (DHF) Department, Children’s Hospital 1, Ho Chi Minh City (Saigon), Vietnam, and Associate Professor of Pediatrics, University of Medicine and Pharmacy, Ho Chi Minh City, died on June 11, 2018. Dr. Lan was born in Thanh Hoa Province on March 29, 1937, educated in secondary schools in Da Lat City, admitted to the Saigon University of Medicine, and graduated with a medical degree in 1965. In 1977, he was appointed to Children’s Hospital 1, where he assumed leadership of the DHF department from 1984 to 1997. Dengue disease comprised a major burden during his tenure, with 20,000–200,000 annual admissions throughout the country. At Children’s Hospital 1, the average annual mortality rate of dengue shock syndrome (DSS) was 12.5%, but through Dr. Lan’s efforts this rate fell to 1.4–0.5%. Because of his intense desire to save lives and teach others his skills, Dr. Lan set in motion changes in clinical and organizational practices that continue to the present time and have brought about steep declines in DHF/DSS fatality rates throughout Vietnam. In 2002, he published a report describing a group of dengue patients often overlooked: severe dengue in infants, 6–11 months of age, born to dengue-immune mothers. These patients develop a more severe disease with higher case fatality rates than do older children. He published a series of classical descriptions of the diagnosis, pathophysiology, and treatment of these cases.

As head of a new DHF department, Dr. Lan recognized that reducing DHF/DSS case fatality rates, particularly during epidemics, required education of mothers as well as specific and continuing training of nurses and physicians. It is crucial to the survival of children that mothers/caretakers recognize warning signs of shock and promptly bring at risk children to hospital. During epidemics, inpatient facilities may be overwhelmed and medical staff can be rapidly exhausted. To avoid an overload of inpatients, Dr. Lan created a dengue unit in the Outpatient Department (OPD) to triage suspected DHF cases. Many suspected DHF cases can be managed as outpatients using oral fluids. However, those outpatients thought to be in impending shock are admitted directly to an OPD emergency department for initial resuscitation. Patients continuing in shock or who have complications (such as respiratory failure or severe bleeding) are transferred to the inpatient intensive care unit. Patients in a stable condition are admitted to the DHF department, where specially trained staff doctors and nurses have access to intravenous fluids, medications, bedside microhematocrit centrifuges, blood products, oxygen, nasal continuous positive airway pressure, central venous lines, and bedside X-ray.

Beginning in 1997, Dr. Lan led colleagues in the design of national standards for DHF treatment. He then organized and conducted training courses for provincial health-care workers at Children’s Hospital 1. A dengue management team was set up in hospitals located in dengue-endemic provinces. The abilities of DHF intervention teams at provincial and referral hospitals throughout Vietnam were assessed annually. Staff were encouraged to consult together for management of severe cases or discuss case management with experienced teams in regional and central hospitals by a hotline that connected health-care facilities by telephone, fax, or e-mail. These measures had a major impact, reducing the fatality of DHF/DSS in Vietnam. During 1964–1974, the DHF/DSS case fatality rate in South Vietnam was 8.26%. Since then, outbreaks of DHF have steadily increased in size, peaking in 1998, when there were 119,429 cases and 342 deaths in 19 provinces of southern Vietnam. Remarkably, during 1999–2006, as training in DHF/DSS case management was extended to all levels of the health-care system, there was a decline in DHF fatality rates to 0.09–0.29% and that of DSS to 0.55–2.0%.

Nguyen Trong Lan was a warm and friendly man, and a devoted husband and father to a large successful family spread throughout the world. He was admired and loved by his students, fellow workers, and those professionals who came to know him through his publications and attendance at international meetings. His legacy will be the many children who because of his efforts have and will survive to live long, happy, and successful lives.

